# Natural Products Targeting Hsp90 for a Concurrent Strategy in Glioblastoma and Neurodegeneration

**DOI:** 10.3390/metabo12111153

**Published:** 2022-11-21

**Authors:** Sarmistha Mitra, Raju Dash, Yeasmin Akter Munni, Nusrat Jahan Selsi, Nasrin Akter, Md Nazim Uddin, Kishor Mazumder, Il Soo Moon

**Affiliations:** 1 Department of Anatomy, Dongguk University College of Medicine, Gyeongju 38066, Republic of Korea; 2 Product Development Department, Popular Pharmaceuticals Ltd., Dhaka 1207, Bangladesh; 3 Department of Clinical Pharmacy and Molecular Pharmacology, East West University Bangladesh, Dhaka 1212, Bangladesh; 4 Department of Pharmacy, Southern University Bangladesh, Chittagong 4000, Bangladesh; 5 Department of Pharmacy, Jashore University of Science and Technology, Jashore 7408, Bangladesh; 6 School of Optometry and Vision Science, UNSW Medicine, University of New South Wales (UNSW), Sydney, NSW 2052, Australia

**Keywords:** Hsp90, glioblastoma, neurodegenerations, chemosensitivity

## Abstract

Glioblastoma multiforme (GBM) is one of the most common aggressive, resistant, and invasive primary brain tumors that share neurodegenerative actions, resembling many neurodegenerative diseases. Although multiple conventional approaches, including chemoradiation, are more frequent in GBM therapy, these approaches are ineffective in extending the mean survival rate and are associated with various side effects, including neurodegeneration. This review proposes an alternative strategy for managing GBM and neurodegeneration by targeting heat shock protein 90 (Hsp90). Hsp90 is a well-known molecular chaperone that plays essential roles in maintaining and stabilizing protein folding to degradation in protein homeostasis and modulates signaling in cancer and neurodegeneration by regulating many client protein substrates. The therapeutic benefits of Hsp90 inhibition are well-known for several malignancies, and recent evidence highlights that Hsp90 inhibitors potentially inhibit the aggressiveness of GBM, increasing the sensitivity of conventional treatment and providing neuroprotection in various neurodegenerative diseases. Herein, the overview of Hsp90 modulation in GBM and neurodegeneration progress has been discussed with a summary of recent outcomes on Hsp90 inhibition in various GBM models and neurodegeneration. Particular emphasis is also given to natural Hsp90 inhibitors that have been evidenced to show dual protection in both GBM and neurodegeneration.

## 1. Introduction

The most prevalent type of primary brain tumor, glioblastoma multiforme (GBM), is among the deadliest forms of human cancer due to its aggressiveness, invasiveness, and neurodestructiveness, with a survival rate of less than 5% [1]. GBM is derived from the glial cells and is accompanied by broad neurological dysfunctions [2]. Despite rigorous improvement in cancer diagnosis and treatments, the curative options in GBM treatment are still limited; thus, finding effective therapies is an immediate concern. Assuredly, conventional strategies, including chemotherapy, surgery, radiotherapy, and immunotherapy, are the potential options that increase the life expectancy of the patients yet are considered insufficient [3,4,5] because GBM tumors are notoriously difficult to exterminate by radical surgery or radiation treatment due to their invasive nature, leading to poor prognosis and repeated recurrences. Furthermore, resistance to chemotherapy is also a more frequent case in GBM therapy [6], whereas the application of radiotherapy is associated with neurodegeneration [7]. Thus, special consideration must be taken when applying classical therapies and incorporating new alternative strategies.

The concern about neurocognitive deficits is becoming more and more critical in neuro-oncological studies, which is the most common complication in long-term survivors [8]. Indeed, tumors in the brain are one of the leading causes of neuronal dysfunction and cognitive declines, similar to those typically detected in neurodegeneration, including mitochondrial damage and synaptic dysfunctions [9,10,11,12]. Even cognitive dysfunction is more pronounced in patients treated with whole-brain radiation therapy alone or with chemotherapy than in untreated patients [9,13,14,15]. Therefore, identifying the new molecular target that implicates the molecular process of GBM proliferation, invasion, chemosensitivity, and neurodegeneration would be a novel strategy to mitigate GBM and GBM-mediated or GBM treatment-induced neurodegeneration.

In this context, Hsp90 (Heat shock protein 90) can be a good candidate target, supported by recent observations highlighting the promising effects of Hsp90 inhibitors in cancer and neurodegeneration. Hsp90 is one of the most abundant proteins in mammalian cells that involves various cellular functions, including protein folding, assembly, trafficking, and clearance [16]. Hsp90 has been considered a potential target in cancer from the beginning and, more recently, in neurological disorders [17,18].

Highlighting the recent evidence on Hsp90 pharmacology, this review discusses that Hsp90 inhibition could signify a potential strategy in GBM therapy by targeting various factors in recurrence, invasion, treatment resistance, and, most importantly, neurodegeneration. In addition, a diverse range of nature-derived Hsp90 inhibitors is also discussed, which are pharmacologically active in both GBM and neurodegeneration, and could be used as potential leads for designing and developing potent Hsp90 inhibitors.

## 2. An Overview of Glioblastoma Multiforme (GBM)

Due to the heterogeneity, GBM tumors are complicated to treat and mainly occur in the brain corticotemporal region, specifically in the cerebral hemispheres (in the subcortical white matter). Nevertheless, it also appears in the spinal cord, brain stem, and cerebellum [19]. Although the term ‘Glioma’ represents tumors originating from glial cells, it is categorized into four types, astrocytic tumors (astrocytoma grades I, II, anaplastic astrocytoma, and Glioblastoma or GBM), oligodendrogliomas, ependymomas, and mixed gliomas [20]. Among all these subtypes, GBM shows exceptional characteristics such as vascular proliferation and necrosis [21]. Based on clinical characteristics, GBM may be primary (usually occurs in patients aged >50 years) or secondary (more common among younger patients) [22,23]. Primary GBM patients have a mean age of 62, whereas secondary GBM patients have a mean age of 45 [24]. Primary GBM is caused by several factors, including overexpression of *MDM2* (Mouse double minute 2), mutation of *EGFR* (epidermal growth factor receptor), *p16* deletion, *TERT* (telomerase reverse transcriptase) promoter mutation, and loss of heterozygosity of chromosome 10q holding PTEN (phosphatase and tensin Homolog) [22,23]. On the other hand, the pathophysiology of secondary GBMs, includes overexpression of several genes (*RB* (Retinoblastoma protein), *PDGFRA* (Platelet-derived growth factor receptor alpha), and its ligand *PDGFA* (Platelet-derived growth factor alpha)), mutation of *ATRX* (Alpha thalassemia X-linked intellectual disability), *IDH1/2* (isocitrate dehydrogenase1/2), and *TP53* (Tumor protein P53), and also loss of heterozygosity (LOH) of 19q [22,23,25]. Additionally, the involvement of different types of mutations makes the pathology of GBM more complex, such as *p53* deletion, upregulation of *VEGF* (vascular endothelial growth factor), *EGFR*, *MDM2,* and *MGMT* (O(6)-methylguanine-DNA methyltransferase) [26]. GBM is more common among men than women, with a ratio of 1.3 to 1 [27].

The etiology of GBM is not entirely known, but like all other cancers, environmental and genetic factors are considered high-risk [28]. The environmental risk factors include ionizing radiation (IR), and some occupational factors include exposure to electromagnetic fields (EMF) [28] and chemical carcinogenesis exposure from the workplace [29]. Primary brain tumors have occurred in many members of the same family in around 5% of GBM cases. In addition, the increased risk of GBM is co-related with other genetic syndromes like Cowden disease, Neurofibromatosis 1 and 2 mutations, Turcot syndrome, and Li–Fraumeni syndrome [30,31].

A GBM tumor is large and irregularly shaped and generally originates in white matter [32]. It is heterogenous and may have a gross appearance with yellow color tissue necrotic areas and regions with cystic degeneration and hemorrhage [33]. As the early symptoms of GBM are prevalent, like headache, confusion, memory loss, speech problems, and seizure, it is impossible to have a diagnosis from these symptoms. The confirmatory diagnosis of GBM is MRI (Magnetic resonance imaging) or CT (Computed tomography), which shows the tumor as a mass surrounded by edema [23,25]. The pattern of the tumor can be focal, multifocal, or diffused [34].

Among the current therapeutic strategies, neuro-surgery is the first choice for GBM treatment [35,36] and reduces tumor size by rebuking. However, owing to its infiltrative growth pattern into neighboring tissue, total tumor excision is clinically tricky [22], and the residual tumor cells persist despite complete resection [30]. Because of that, surgery, chemotherapy, and radiotherapy are required to manage disease progression. Again, the administration of chemotherapy is limited due to the blood-brain barrier (BBB). Temozolomide (TMZ), an alkylating agent, is the first choice in this case [37,38]. Generally, in the post-operative stage, a combination of radiation and TMZ prolonged the survival rate. In the case of post-operative treatment, radiation therapy of 60 GY in fractionated dose is conducted over a 6-week time period. It has been reported from phase 3 clinical trials that radiotherapy after surgery can prolong survival time by up to 9–10 months for the patient [39,40].

## 3. Involvement of Hsp90 in Glioblastoma Multiforme (GBM) Progression

**Figure 1:**
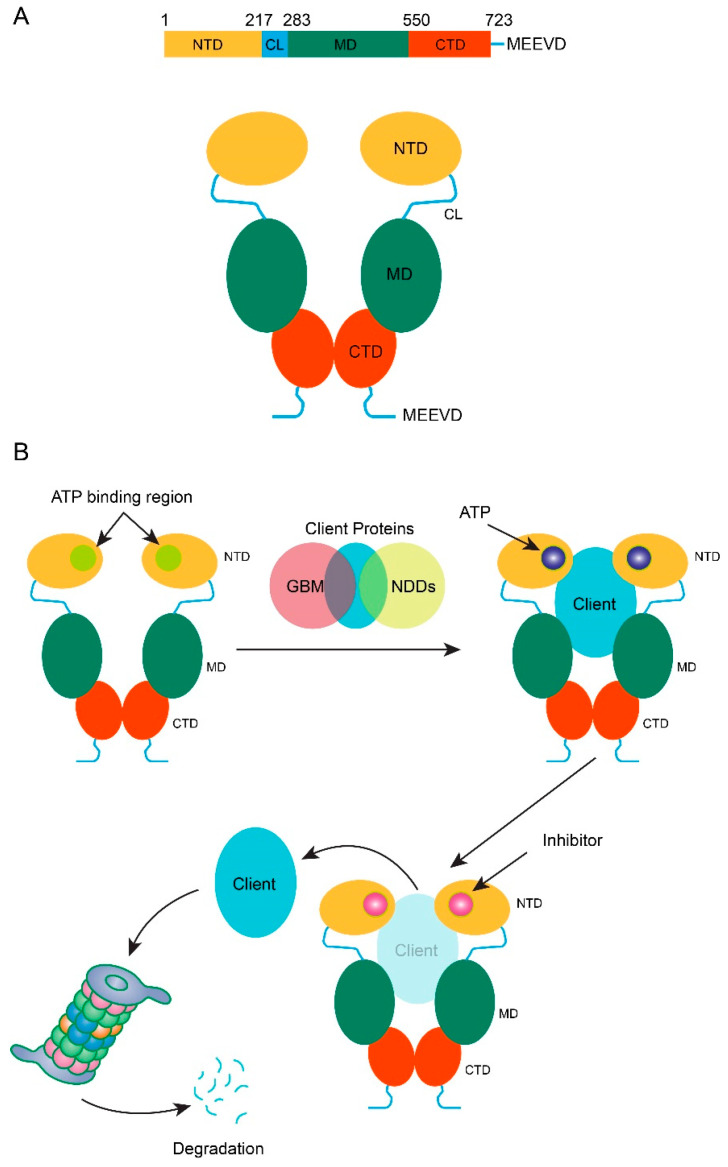
Structure and mechanism of Hsp90. (**A**) Residue mapping showing the structural domains of an Hsp90 monomer and the structure of an Hsp90 dimer. (**B**) General mechanism of Hsp90 in stabilizing and degrading client proteins (causative factors in GBM and NDDs). Client protein Hsp90 binding to the various client protein is facilitated by a conformational change in dimers induced by the binding of ATP in the NTD domain. Hsp90-targeted inhibitors compete with ATP and cause a release of client proteins leading to the degrading by the ubiquitin-proteasome degradation system. NTD, N-terminal domain; CL, charged linker region; MD, middle domain, CTD, C-terminal domain; ATP, adenosine triphosphate; GBM, glioblastoma multiforme; NDDs, neurodegenerative diseases.

Hsps (Heat shock proteins)are a vast family of molecular chaperones that play various functions in cellular maintenance and development by controlling protein folding, maturation, and destruction [41]. This protein family can be divided into small ATP (Adenosine triphosphate)-independent and large ATP-dependent Hsps. Large ATP-dependent HSPs range in size from 40 to 105 kDa. Among them, one of the most important members is Hsp90, consisting of 90 kDa molecular weight [42]. There are five functional Hsp90 members identified to date in mammalian cells, including the major cytoplasmic isoforms, Hsp90a1/a2 and Hsp90b, and GRP94 (glucose-regulated protein 94) in the endoplasmic reticulum (ER), and TRAP-1 (Tumor necrosis factor receptor-associated protein 1) in mitochondria [43]. Additionally, it is involved in essential cellular processes like DNA repair, development, and immune responses [44]. Hsp90 shows elevated cellular expression due to cell damage and regulated large numbers of proteins via the maturation process and folding [45]. Structurally, Hsp90 is composed of three domains, including a middle domain (MD), an N-terminal ATPase domain (NTD), and a carboxy-terminal domain (CTD) (Figure 1A) [46,47,48], which operate as binding sites for client proteins and co-chaperones (Figure 1B), and thus can interact with more than 20 co-chaperones and regulate their biological activity. As it mediates many proteins, Hsp90 plays a significant role in various signal transduction pathways involving the development of numerous oncogenesis processes and progression. This phenomenon makes Hsp90 a suitable target for treating severel cancers. Many studies target Hsp90 inhibition and design Hsp90 inhibitors to treat different cancers, especially in GBM [49].

There is a close relationship between Hsp90 and GBM development, as Hsp90 promotes metastasis, inhibits apoptosis, and helps develop resistance to chemo and radiotherapies [50,51]. Studies showed that Hsp90 interaction with its client proteins significantly improves the viability of GBM cells. Client proteins include MAPK (mitogen-activated protein kinase), hTERT (human telomerase reverse transcriptase), FAK (focal adhesion kinase), PDGFR (Platelet-derived growth factor receptor), PI3K (phosphoinositide-3 kinase), p53, CDK4 (cyclin-dependent kinase 4), AKT (protein kinase B), EF-2 kinase (elongation factor 2), HIF-1a (hypoxia-inducible factor 1alpha), mutated variant EGFRvIII (epidermal growth factor receptor variant III), and EGFR (epidermal growth factor receptor) [52]. The common pathways in GBM cell proliferation, i.e., PI3K/Akt and MAPK, are also known to modulate by Hsp90 [53]. Grade III and IV brain tumors (up to 63%) evidence the presence of an alteration in one of four genes, including *PIK3CA* (Phosphatidylinositol-4,5-Bisphosphate 3-Kinase Catalytic Subunit Alpha), PIK3R1 PTEN, and EGFR in PI3/Akt signaling pathway [54], whereas these genes are dependent on the Hsp90 clients, such as mutant EGFRvIII and other essential kinases [43]. Recent work has also shown that in U87MG human GBM cells, activation of EGFR can elevate VEGF(vascular endothelial growth factor) expression by transcriptional activation of the VEGF promoter via a PI3K-dependent pathway [55]. VEGFR1 (vascular endothelial growth factor receptor 1) and VGFR2 (vascular endothelial growth factor receptor 2) are both client proteins of Hsp90 [56], suggesting that Hsp90 inhibitors can inhibit VEGFR production by tumor cells to block cell proliferation.

GBM shows a high level of inherent radioresistance derived from the overexpression of *DDR* (DNA damage response) genes and basally increased DDR activity. Interestingly, many DDR regulators depend on Hsp90 for protein folding [57,58]. Again, Hsp90 modulates some client proteins to suppress apoptosis in cancer cells. Hsp90 protects inhibitor proteins of apoptosis. A well-known member of apoptosis, p53 is a tumor-suppressive protein that regulates normal and tumor cell growth by apoptosis or cell cycle arrest. Mutant p53 may cause dysregulation of apoptosis as well as cell proliferation. Hsp90 makes a complex with a specific mutant of p53 (temperature sensitive) in the cytoplasm [59]. Another mutant, p53, makes a stable complex with Hsp90 [60]. Many tumor-inducing p53 mutations may increase the binding affinities of p53 to Hsp90. Some other apoptotic proteins, Bcl2 (B-cell lymphoma 2) and Bcl-xL (B-cell lymphoma-extra-large), also interact with Hsp90 [61,62]. Therefore, the apoptotic pathway in the cancer cell is dependent on Hsp90.

Collectively, blocking Hsp90 ATPase activity by using specific inhibitors can be an attractive strategy for overcoming the limitations of conventional GBM treatment, as many Hsp90 client proteins are involved in the GBM development and maintenance, including CyclinD1, CDK4, Akt, and EGFR. Inhibiting Hsp90 will stimulate the degradation of Hsp90 client proteins by ubiquitin-proteasome systems and concurrently can multiply targets in various signal transduction pathways that promote GBM proliferation and survival [63,64].

## 4. HSP90 Inhibition in the Aggressiveness of Glioblastoma Multiforme (GBM)

The high recurrence rate and innate resistance to chemotherapy and radiation for GBM earn it the title “most aggressive brain tumor”. As described previously, critical contributing factors are DDR amplification, and adaptive mechanisms play a vital role in this context [1,65,66,67,68,69]. Intriguingly, the treatment of NW457, a second-generation Hsp90 inhibitor in GBM cells, has been shown to reduce the expression of DDR regulators with limited cytotoxicity at low nanomolar doses. In addition, in an in vivo mouse model of GBM, Hsp90 suppression synergistically lowers tumor development and survival after radiation therapy (CBCT-based irradiation) while also decreasing irradiation-induced GBM cell migration and tumor invasiveness [70]. Combined treatment with the Hsp90 inhibitor NXD30001 and radiation dramatically prolonged median life and decreased tumor volume in mice with GBM, which resulted from the impairment of DDR by changing DDR-related protein expression [71]. Previously, Choi et al. found that increased radio-sensitizing effects in gliomas are associated with reversing EMT (epithelial-mesenchymal transition), inducing apoptosis or autophagy, and impairing DNA damage repair, which could be achieved by using Hsp90-specific inhibitors via modulating PI3K and mTOR (mammalian target of rapamycin) pathway [72]. Similarly, Sasame et al. showed that inhibition of Hsp90 in glioma (high-grade, *BRAF* V600E-mutant) effectively overcame the resistance. It was found that apoptosis was induced by the dephosphorylation of GSK3β (Ser9) (glycogen synthase kinase-3 beta serine 9), and suppression of Bcl-2 family members after Hsp90 inhibition in combination with BRAF/MEK inhibition, which also deactivated MAPK and AKT/mTOR pathways [73]. The same is also shown by other studies [74]. Using cell culture and xenograft models, Sun et al. showed that GBM cell proliferation was reduced when the cells were treated with an Hsp90 inhibitor, NMS-E973, by upregulating the proapoptotic Bcl-2 member PUMA in a p53-dependent way. Moreover, NMS-E973 therapy induced xenograft tumors and a lack of PUMA greatly attenuated the anticancer effects of NMS-E973 [75]. Inhibitors of Hsp90, such as Hsp90, and PI3K, such as BKM120, both with and without RT, are effective against GBM cells and tumors Wachsberger et al. suggesting that their efficacy is independent of PTEN/p53 status [76].

A mitochondria-targeting Hsp90 inhibitor, gamitrinib, has decreased tumor development and increased mouse survival in subcutaneously and intracranially implanted patient-derived and cell line-derived xenografts—tumor models. Furthermore, the growth of neurospheres, patient-derived organoids, TMZ-resistant glioma cell lines, and primary glioma cell lines was suppressed, and apoptosis and cell death were promoted by gamitrinib treatment. Mechanistically, gamitrinib inhibits cell-cycle progression, OXPHOS, and mitochondrial biogenesis while inducing DNA damage, energy-sensing AMP-activated kinase, and stress response [77]. A mechanistic study by Ho et al. showed that N-vinylpyrrolidone-AUY922, an Hsp90 inhibitor, can promote cell death of patient-derived GBM cells by downregulating NF1 (Neurofibromin), PDGFRA, EGFR, and CDK4 expression while inducing apoptosis and autophagy [78]. Recently Li et al. showed that the FGFR3-TACC3 (F3-T3) fusion gene, a client of Hsp90, was found highly in both untreated and matched recurrent GBM during concomitant irradiation and TMZ treatment. Sensitization of F3-T3 glioma cells to TMZ was achieved by inhibiting Hsp90, which reduced F3-T3 activation and increased TMZ-induced DNA damage [79]. There is substantial evidence that Hsp90 is a critical element in GBM cell migration and invasiveness. It has been found that blocking Hsp90 activity reduces migratory potential in DK-MG and SNB19 GBM cell lines by interfering with the signaling pathways of its clients AKT and MEK. These anti-migratory effects have been linked to a change in the cytoskeleton [80].

Glioma stem cells (GSCs) are a vital element contributing to treatment resistance in GBM, and multiple investigations have shown that *HSP90AA1* (Heat Shock Protein 90 Alpha Family Class A Member 1) inhibitors successfully induce cell death in GSCs. Using spheroids produced from T98G cells, Tani et al. analyzed the radiosensitizing effects of N-vinylpyrrolidone-AUY922, on SCPCs and spheroid CD133-negative cells (SCNCs). When comparing CD133+ and CD133− cells, NVPAUY922 increased radiosensitivity in the former more than in the latter, suggesting a promising radiosensitization candidate for GBM cancer stem cells [81]. GSCs with a high capacity for homologous recombination (HR) DNA repair are more resistant to chemoradiation. A previous study showed that onalespib treatment sensitized GSCs to the combination of radiation and TMZ by depleting CHK1 (Checkpoint kinase 1) and RAD51 (RAD51 Recombinase), two critical proteins of the HR pathway and attenuating HR repair (TMZ). Inhibiting Hsp90 reconfigured the transcriptome of GSCs and significantly changed the expression of cytoplasmic proteins, including both well-characterized and new client proteins. In a zebrafish and a mouse xenograft model of GBM, the addition of onalespib to the standard of therapy (radiation and TMZ) resulted in significantly longer lifetimes than either radiation alone or onalespib plus radiation [82]. In combination with TMZ, another blood-brain barrier crossing Hsp90 inhibitor, onalespib showed a decrease in angiogenesis, migration, proliferation, and viability of patient-derived glioma-initiating cells and GBM cell lines by depleting several survival-promoting client proteins such as AKT, EGFRvIII, and EGFR, extended survival in several in vivo models [83]. Taken as a whole, inhibition of Hsp90 decreases heterogeneous oncogenic markers of GBM and increases radiation/chemosensitivity.

## 5. Implication of Glioblastoma Multiforme (GBM) in Neurodegeneration

Patients of GBM show a variety of neurological symptoms as a result of progressive neurodegeneration [84,85,86], which depends on the localization of the tumor, its expansion, subsequent intracranial pressure, and edema [87]. Using the autopsy brains of patients with GBM, Nelson et al. confirmed the hallmarks of neurodegeneration in 42% percent of cases of GBM [88]. However, other studies have pointed out that aging can contribute to neurodegeneration and brain tumor formation [89]. In most cases, adult patients with brain tumors, especially those with slow-growing, low-grade, or benign tumors like ganglioglioma or meningioangiomatosis, have been shown to contain protein deposits linked with neurodegeneration, such as neuropil threads, neurofibrillary tangles (tau-positive), and granulovacuolar degeneration [90,91,92,93,94,95]. Ricken et al. also identified that the cells in GBM tumor and tumor-neighboring tissues exhibit increased autophagy in an autopsy-based study, and concluded that neurodegenerative protein aggregate accumulation in GBM is more likely due to age-related, asymptomatic diseases [89].

On the other hand, several recent studies reported various links between GBM and neurodegenerative disease. Several animal models have shown that GB-caused progressive neurodegeneration may be the primary basis for neurological symptoms [85,86]. In a Drosophila model, a molecule is identified that is involved in the insulin pathway, named ImpL2 (Ecdysone-inducible gene L2), generated from GB cells, and impacts neuronal function like mitochondrial defects or synapse loss. However, restoring insulin signaling in neurons may inhibit tumor advancement in *Drosophila* models [96]. In addition, synapse number is reported to be significantly reduced in the motor neurons of the *Drosophila* model upon GB induction [86]. WNT signaling pathway plays a significant role in GB progression, and it also plays a crucial role in brain development [97], adult neuronal physiology [98], and synaptogenesis [99]. WNT signaling dysregulation is associated with neural deficits such as Alzheimer’s disease (AD) [100,101]. The WNT (Wnt Family Member 1) signaling pathway is frequently upregulated in GB [102], explaining the link between GB and resulting neurodegeneration. Again, abnormal phosphorylation and aggregation of Tau are pathologically related to neurodegenerative diseases [103] like AD, corticobasal degeneration, and progressive supranuclear palsy [104]. Soluble CD44 has been identified to induce Tau-phosphorylation and subsequent neurodegeneration. In the case of 58% of gliomas, an elevated level of soluble CD44 is detected [105]. Initiation of tau pathology in the brain by sCD44 and overexpression of CD44 in glioma represents the pathological relation between GBM and neurodegeneration [106].

Loss of the proteostasis network is the main pathological remark of many neurodegenerative disorders and is caused by overexpression of pathological misfolded proteins, excessive ROS (reactive oxygen species), neuroinflammation, ER stress, and mitochondrial dysfunction. One of the major degradation systems of the proteostasis network, autophagy, which is dependent on the mTOR signaling pathway, is usually identified to be suppressed in GBM cells due to the overactivation of the mTOR signaling pathway [107]. Interestingly, Ryskalin identified that this suppression of autophagy induced the aberrant accumulation of α-synuclein (α-syn) aggregate, a marker of Parkinson’s disease (PD), in GBM cells more than usual astrocytes, which are hardly degradable by Proteinase K [108]. Again, activated microglia are thought to assist the GBM during tumor growth, even though they initially deploy their immune machinery to halt the malignant process [109,110]. Studies show that macrophages and activated microglia critically promote the transportation of various signaling molecules and growth factors to transformed astrocytes and adjacent neurons [111,112]. On the other hand, microglial activation is responsible for secreting various proinflammatory cytokines and ROS, which might trigger inflammatory conditions and ER stress in neighboring tissue and neurons, leading to degeneration.

IR is one of the most standard treatment strategies routinely applied in GBM patients; however, during the process area around tumors is also irradiated as part of the therapy process. There was strong evidence that IR exposure exacerbates the stress response in cells and triggers DNA repair errors, affecting cell cycle progression and survival in normal cells [113,114,115]. IR induces ROS by disrupting mitochondria, reducing mitochondrial synthesis, and disrupting calcium homeostasis [116,117,118,119,120,121,122,123]. These changes in signaling pathways triggered an extreme shift in the proteostasis network, which is linked to autophagy deficiencies, protein aggregation, and apoptosis (reviewed in [7]).

Although more studies are necessary to elucidate the exact mechanism, GBM is a direct or indirect inducer of neurodegeneration and other brain disorders. However, GBM accelerates pre-existing hallmarks of neurodegeneration and aging in adult patients, or patients can acquire degenerative processes during GBM therapies, such as radiation. Thus therapies that target GBM but are co-opted for neurodegeneration could become a novel approach for GBM patients.

## 6. Inhibition of Hsp90 in Neurodegeneration

Hsp90 plays a critical role in clearing protein aggregates, folding, and maintaining proteostasis balance. In certain tumors, the Hsp90 chaperone network promotes disease progression by stabilizing various oncogenic client proteins, and on the contrary, evidence suggests that Hsp90 and its co-chaperones control most neurodegenerative proteinopathies [124,125,126,127]. HSF1 (heat shock factor 1), where transcriptional activity is required for the upregulation of chaperone components, remains inactive in basal conditions due to forming a complex with Hsp90 [128,129,130,131]. When stress, such as misfolded protein accumulation, is induced, Hsp90 releases HSF1, which expresses various chaperones, including Hsp70 (heat shock protein 70) [128,132] and other antioxidant proteins, thus promoting misfolded protein aggregates clearance and reducing oxidative stress [133,134,135,136,137,138]. Many small-molecule Hsp90 modulators that antagonize the Hsp90-HSF1 complex have been developed in recent years, and many of them are analyzed for their neuroprotective properties in the various neurodegenerative properties. Combining the geldanamycin derivatives (tanespimycin, 17-AAG, a semisynthetic Hsp90 inhibitor) with the nanoparticle HMPB (Hollow mesoporous Prussian blue) resulted in remarkable therapeutic effects on tau-related pathologies in both in vivo and in vitro experiments. In vitro studies proved that HMPB combination with AAG could lower p-tau levels, blocking ROS generation and bolstering neuroprotection against apoptosis. In the okadaic acid (OA)-induced AD rat model, HMPB-AAG nano-agents ameliorated memory impairments and tau-related pathologies by boosting Hsp40/Hsp70-mediated p-tau degradation, reducing oxidative stress, mitigating neurotoxicity, and preventing neuronal death and synaptic loss [139]. Similarly, substantial evidence from experiments demonstrates that ROS produced as a byproduct of dopamine metabolism, low glutathione (GSH), and high levels of iron and calcium in the SNpc is considered an essential factor in the death of dopaminergic neurons in the PD brain [140]. Additionally, 17-AAG was found to lower alpha-synuclein toxicity and promote alpha-synuclein removal by inducing autophagy in several PD models [141,142,143]. It was also found to be cytoprotective in another study, resulting in lower levels of mutant SOD1 in primary neurons [144]. Fujikake et al. reported that 17-AAG could inhibit polyglutamine-induced neurodegeneration in an HSF1-dependent manner in *Drosophila melanogaster* models of the polyQ disorders, including HD and spinocerebellar ataxias (SCA) [145]. Androgen receptor (AR), an Hsp90 client protein, is a misfolded protein that tends to congregate in neurons in spinal and bulbar muscular atrophy (SBMA). Administration of HSP90 inhibitor, 17-(dimethylaminoethylamino)-17 demethoxygeldanamycin (17-DMAG) at 10 mg/kg lowered levels of monomeric and nuclear-accumulated mutant androgen receptor (AR) by increasing proteasomal degradation and dramatically improved motor deficits in SBMA animals without causing noticeable toxicity [146]. Androgen receptor (AR), an Hsp90 client protein, is a misfolded protein that tends to congregate in neurons in spinal and bulbar muscular atrophy (SBMA). Administration of Hsp90 inhibitor, 17-(dimethylaminoethylamino)-17 demethoxygeldanamycin (17-DMAG) at 10 mg/kg lowered levels of monomeric and nuclear-accumulated mutant AR by increasing proteasomal degradation and dramatically improved motor deficits in SBMA animals without causing noticeable toxicity [146]. Machado–Joseph disease (MJD) is the most prevalent inherited spinocerebellar ataxia and a polyglutamine-based neurodegenerative disorder [147]. The numbers of ataxin-3 nuclear inclusions were reported to reduce in 17-DMAG-treated (25 mg/kg) CMVMJD135 transgenic mice and displayed normal cell shape, with the absence of abnormally small cells in this area of the brain compared to transgenic vehicle-treated animals. Authors also found the increased level of the Beclin-1 protein in the brainstem of 16- and 30-week-old mice, as well as the microtubule-associated protein, LC3-II/LC3-I ratio suggesting the activation of autophagy by continuous 17-DMAG therapy in the reduction in neuropathology [148]. Putcha et al. had paid an effort to reduce the level of α-syn aggregation by using the Hsp90 inhibitor, SNX-0723 (2-fluoro-6-[(3S)-tetrahydrofuran-3-ylamino]-4-(3,6,6-trimethyl-4-oxo-4,5,6,7-tetrahydro-1H-indol-1-yl) benzamide). It was shown that SNX-0723 (48nM) had an EC_50_ for suppression of α-syn oligomerization and may reverse α-syn-induced toxicity. Based on in vivo testing, SNX-0723 was found to have high concentrations in the brain six hours after an oral administration (10 mg/kg) and to induce brain Hsp70, suggesting that it might be used in an oral treatment regimen [141]. In the study by Luo et al., inhibiting Hsp90 by a synthetic small molecule PU-DZ8 (75 mg/kg) was observed to reduce both soluble and insoluble tau aggregate accumulation in tauP301L female mice [149]. Furthermore, single-cell transcriptomics shows that Hsps are responsible for the neurodegenerative process and its accompanying reactive astrogliosis (GFAP). After therapy with PU-H71, the malfunctioning Hsp epichaperome was removed, reducing the reported tau pathology and transcriptional responses [150]. The administration of blood-brain barrier–permeable Hsp90 inhibitor EC102 (200 mg/kg per day) for seven days led to a significant decrease in the number of abnormal p-tau species (nearly 50%) that were phosphorylated at S396/S404 and S202/T205 but the normal tau species, which ranged from 45 to 55 kDa, were largely unaffected by the EC102 treatment [151]. Reducing sensory neuron loss after axotomy can be achieved by upregulating the expression of Hsp70 [152]. In vitro, KU-32, novobiocin-based, C-terminal inhibitor of Hsp90 delayed the pathological course of (diabetic peripheral neuropathy) DPN in Sprague Dawley rats, and it reduced the amount of neurodegeneration that occurred in myelinated and nonmyelinated sensory neurons by increasing Hsp70 at 10 nM [153]. Furthermore, KU-32 reduced amounts of superoxide in mitochondria, and increased respiratory activity in hyperglycemically stressed neurons at 1 μM [154]. In the 5-fluorouracil (5-FU)-induced cognitive impairment model, KU32 was observed to remove temporal discrimination deficits when administered orally before 5-FU injections [155].

## 7. Natural Products Targeting Hsp90

**Figure 2:**
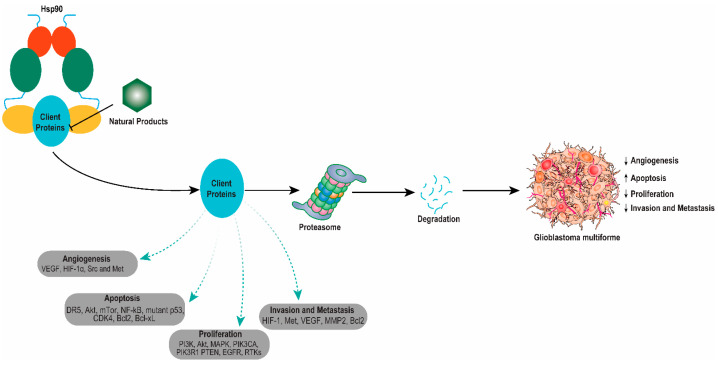
Molecular mechanism of Hsp90 inhibition by natural products in GBM. Hsp90 client proteins play essential roles in GBM progression and maintenance. Targeting several signal transduction pathways that support GBM growth and survival can be facilitated when Hsp90 is inhibited, stimulating the degradation of Hsp90 client proteins by ubiquitin-proteasome systems.

**Figure 3:**
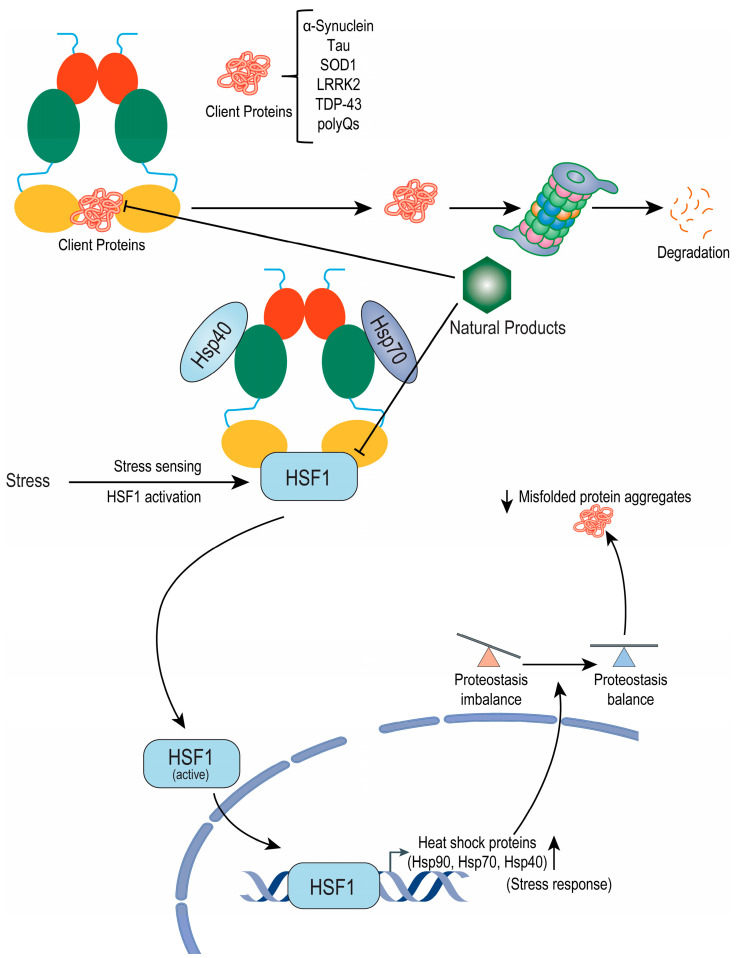
Molecular mechanism of Hsp90 inhibition by natural products in alleviating neurodegeneration. Hsp90 inhibition by natural products could initiate two different processes, resulting in the degradation of client protein (pathological misfolded protein) and improving proteostasis. Hsp90 inhibition causes the release of pathogenic “client” proteins for proteasomal degradation, where the interaction of Hsp90 provides structural stability and maintenance of these client proteins. The inhibition of Hsp90 also stimulates the upregulation of other heat shock proteins via an HSF1-dependent mechanism. These HSPs can improve cell survival in a “stressed” environment by facilitating the clearance and blocking the accumulation of abnormal clients.

**Figure 4:**
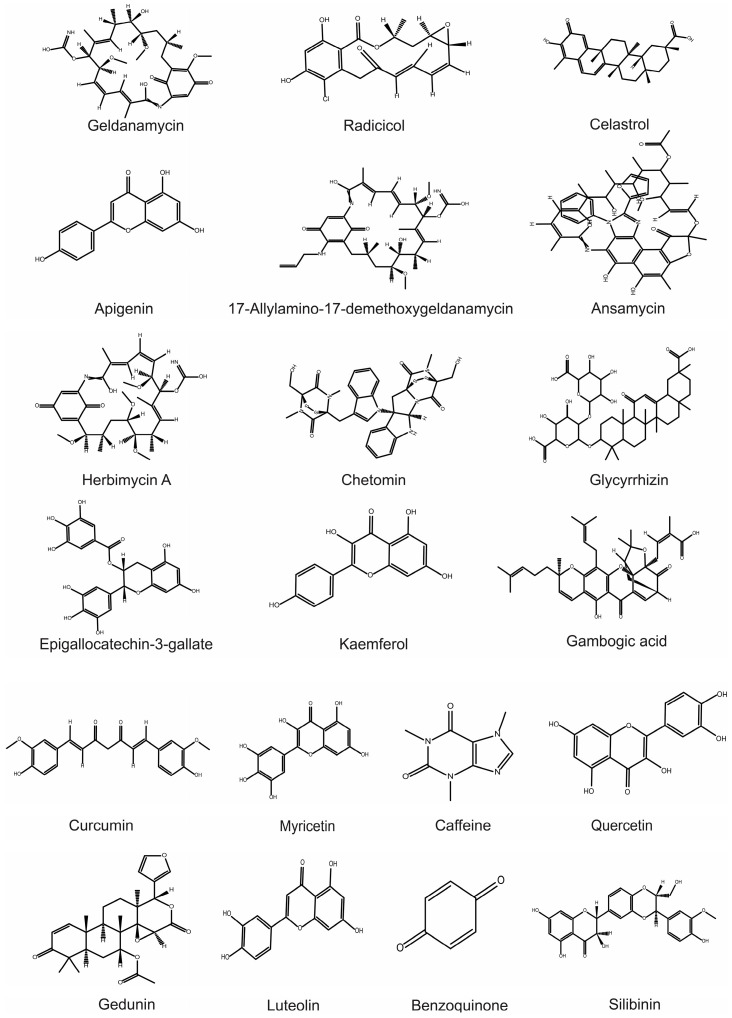
The chemical structures of naturally occurring Hsp90 inhibitors are pharmacologically active in GBM and neurodegeneration.

From the ancient history of drug discovery, nature has been an excellent source of potent leads for developing new drugs against diverse diseases. Similarly, for Hsp90 inhibition, benzoquinone ansamycins were first identified to inhibit Hsp90 by directly binding to the ATPase binding site in the N-terminal domain, which was first isolated from the actinomycetes (*Streptomyces hygroscopicus* species) found in the soil [156]. Several benzoquinone ansamycins, such as geldanamycin and herbimycin A, have received much attention because of the multifaceted assault it provides against cancer by inhibiting apoptosis, oncogenesis, cell cycle progression, and cell proliferation by inhibiting Hsp90 functions [157]. Indeed, several Hsp90 inhibitors are designed based on geldanamycin subjected to more than twenty ongoing clinical studies in the treatment of various malignancies [158]. Thanks to recent developments in bioanalytical methods, DNA sequencing, and molecular modeling, which have sped up the discovery of potent Hsp90 inhibitors from natural sources, very few have been studied extensively for their potential efficacies against both cancers (Figure 2) and neurodegenerations (Figure 3). As many good pieces of literature reviewed the available natural Hsp90 inhibitors [159,160,161], mainly in cancers [159] or neurodegeneration [162], this review will be more engaged in discussing natural Hsp90 inhibitors that already evidenced their potentiality in GBM and neurodegeneration (Figure 4) [163].

### 7.1. Quinone

Pastvova et al. analyzed the effect of geldanamycin (GDN) and 17-allylamino-17-demethoxygeldanamycin (17-AAG, tanespimycin) in various cell lines of GBM. They found that, at sub-micromolar and nanomolar doses, geldanamycin and 17-AAG produced extensive cell death in all GBM cell lines tested, with the morphological and biochemical markers of apoptosis [164]. GDN binds with the ATP binding site of NTD for proteasomal degradation of the Hsp90-client protein complex by E3 ligase [165] and induces autophagy by IKK (IkappaB kinase) degradation, which ultimately blocks the NF-kB (nuclear factor kappa-light-chain-enhancer of activated B cells) signaling pathway [166]. It also inhibits huntingtin aggregation via activation of HSF1, which induces the expression of HSPs (Hsp70, Hsp40, and Hsp90) [167].

The effects of herbimycin A on GBM cell apoptosis were identified by Sonoda et al., where the mechanism was involved in apoptosis acceleration by inhibiting the phosphorylation of FAK kinase [168]. Hamasuna et al. showed that herbimycin A could reduce the growth factors mediated GBM invasion by inhibiting the activation and expression of MMP-2 (matrix metalloproteinase-2) [169]. Herbimycin A is used as a protein tyrosine kinase inhibitor and v-Src transformation following degrading Hsp90-v-Src complex [170,171]; it also causes Hsp90 client protein cell death by upregulating levels of β-catenin and suppressing P13K/AKT signaling pathway [172]. Pharmacological inhibition of Hsp90 by herbimycin A reduces the aggregation of mutant PKCγ (protein kinase C gamma, a common mutant of neurodegenerative disorders) and apoptosis and promotes dendritic development in primary cultured Purkinje cells through the upregulating HSP70 expression [173].

### 7.2. Polyphenol

Epigallocatechin-3-gallate (EGCG) is one of the significant catechins derived from green tea [174]. According to reports, EGCG inhibits the expression of Hsp90 and its client proteins in MCF7 cancer cell lines [175]. EGCG binds at the C-terminal of Hsp90 and inhibits the dimerization of Hsp90 [176]. Additionally, EGCG shows anti-proliferative activity on brain tumor cells [177,178]. Udroiu et al. identified clastogenic of EGCG as its treatment in low doses against radioresistant GBM cells (U251), which resulted in increased telomere shortening-induced senescence and genotoxicity that is independent of telomere length [179]. Das et al. showed that EGCG could selectively induce apoptosis through stimulating ROS generation following JNK (c-Jun N-terminal kinase) activation and MAPK phosphorylation in GBM cells but not in normal astrocytes [180]. Xie et al. showed that GBM cells, but not normal glial cells, are the primary targets of EGCG-mediated MGMT inhibition and TMZ cytotoxicity enhancement [181]. EGCG is also effective in showing neuroprotective against various neurodegenerative diseases, reviewed in [182,183,184,185]. Notably, EGCG, in combination with radiation treatment, was found to reduce irradiation-stimulated DNA damage and apoptosis of hippocampal neurons in rat models supplied with γ-radiation (4 Gy) [186].

On the other hand, a common natural polyphenol, curcumin, is also known for its cytotoxicity against GBM and modulates some common pathways that are misregulated mostly during the GBM progression, including NF-κB, JAK/STAT, P13K/AK, p53, SHH (Sonic Hedgehog Signaling Molecule), and Rb pathways, reviews in [187,188,189]. In addition, curcumin has been shown to make GBM cells more vulnerable to irradiation [190,191] and chemotherapy [192] while protecting normal cells from radiation-induced injuries [193,194,195,196]. Curcumin is also a lead compound of Hsp90 inhibitor [197,198,199,200] suppressed protein aggregation by activating UPR and HSF1 formation in heat-stressed rats in vivo model [201,202,203,204,205]; downregulating Akt/mTOR/p70S6K and activating ERK1/2 signaling pathways induces autophagy [206]. In the case of chronic myeloid leukemia, curcumin is reported to inhibit Hsp90 [207]. In addition, in the case of multiple myeloma, curcumin treatment downregulates the expression of Hsp90 [208].

Caffeine has also been reported to suppress Hsp90 expression and stimulate degradation of Raf-1 (RAF proto-oncogene serine/threonine-protein kinase), Ras, and Akt/PKB via ubiquitin-proteasome systems [209] and showing protective effects by elevating LC3-II (microtubule-associated proteins 1A/1B light chain 3B) expression and activating AMPK in AD and PD disease models [210,211]. In GBM cells, the treatment of caffeine reduces cell proliferation by modulating MAPK signaling and expression of the proteolytic enzymes [212] while invasion by blocking IP3R3-mediated Ca^2+^ release [213] and HIFs/VEGF pathways [214]. By inhibiting G2 arrest, caffeine increases GBM cells (U87-MG) more sensitive to TMZ, which causes mitotic catastrophe and cell death [215].

Radicicol is reported to suppress HGF (Hepatocyte growth factor)/Met-mediated protein network by inhibiting the invasion and migration of GBM cells through Hsp90 inhibition [216]. Through blocking TNF (Tumor necrosis factor)-release and reducing iNOS (inducible nitric oxide synthase) expression in microglia, radicicol conversely protects against LPS (Lipopolysaccharides)/IFN (Interferon)—induced neuronal cell death in neuron-glia cultures [217].

### 7.3. Triterpenoid

Gedunin is a natural triterpenoid compound isolated from *Azadirachta indica*. Gedunin is an Hsp90 inhibitor that degrades Hsp90-dependent client proteins [218]. In the case of GBM, gedunin is reported to show an antiproliferative effect. Gedunin reduces cell proliferation and induces apoptosis in GBM cell lines by attenuating key survival signaling pathways like Akt/mTOR, NF-kB, and apoptotic markers such as PARP (poly(ADP-ribose) polymerase), caspases, and Bcl-xL [219]. In Neuro-2a cells, treatment of gedunin reduced the aggregation of exogenously expressed mHTT and reduced inclusion bodies in fibroblasts from HD patients and neurons derived from induced pluripotent stem cells from patients in a dose- and time-dependent manner. Mechanistically, gedunin promotes the clearance of aggregates depending on the proteasomal pathway rather than the autophagy route [220]. Rane et al. demonstrated the neuroprotective of gedunin, which reduced Aβ (Amyloid β-protein)_1–42_ oligomer-induced neuroinflammation and microglial reactivity by inhibiting NF-kB activation while activating Nrf2 (Nuclear factor erythroid 2-related factor 2) [221].

A natural triterpene compound, celastrol extracted from *Tripterygium wilfordii*, is known to have anti-cancer properties via the Hsp90 degradation mechanism [222]. Celastrol ameliorates ATPase activity of Hsp90 without blocking ATP binding resulting in Hsp90 client protein degradation [223], and enhances expression of HSPs by acting on HSF1 [224] and degenerate mutant protein accumulation through suppressing NF-kB activation and BACE-1 (Beta-secretase 1) expression [225]. Celastrol has also been found to inhibit CDC37 (cell division cycle 37) and Hsp90 interaction [226]. Celastrol regulates the chaperone activity of Hsp90 by binding to its C-terminal and N-terminal domain and disrupts Hsp90-cdc37 involute [227]. Celastrol is pharmacologically active in showing its neuroprotective effect in PD [228] and HD [229] by inducing Hsp70 expression, regulating autophagy and mitophagy, and reducing neuroinflammation and oxidative stress [230,231]. Studies have also reported that celastrol inhibits GBM cell proliferation and induces apoptosis [232,233]. Specifically, Boridy et al. found that celastrol induced apoptosis in GBM cells by regulating the proteostasis network, such as inducing expression of Hsp90 and Hsp72, and autophagy substrate, P62 (sequestosome 1), suggesting that by inducing proteotoxic stress, celastrol promotes cell death [234]. An investigation by Cheng et al. showed that celastrol is effective in inducing apoptosis in GBM cells by modulating DR5 in the death receptor pathway [235], whereas He et al. suggested that celastrol triggered ER stress-regulated apoptosis by inhibiting the PERK/Qrich1 pathway [236].

Another compound, glycyrrhizin, as a co-inducer, reported dissociating the Hsp90-glucocorticoid receptor complex and enhancing Hsp expression [237,238,239]. Glycyrrhizin provides neuroprotection by enhancing endogenous antioxidant defenses, reducing excitotoxicity induced by Glutamate, and attenuating neuron injuries [240]. In the zebrafish model of PD, glycyrrhizin treatment reduced inflammation and apoptosis by regulating autophagy [240]. Further reports also suggested that glycyrrhizin NLRP3 inflammasome [241] reduces microglial activation and thus provides neuroprotection in various CNS injuries [242,243,244]. Although many pieces of literature report the anticancer activity of glycyrrhizin, the effect in the GBM model is scared. Only a recent study by Gao et al. showed an enhanced growth-inhibiting action of TMZ by treating glycyrrhizin using in vivo xenograft GBM model [245].

### 7.4. Xanthone

Gambogic acid extracted from gamboge, the brownish dry resin of the Southeast Asian Garcinia hanburyi tree, is known to inhibit Hsp90 by binding directly to ATP binding site [246]. Thida et al. showed that gambogic acid could inhibit GBM cells dose-dependently by inducing ROS and apoptosis signaling pathways [247]. Qiang et al. showed that gambogic acid could inhibit the growth of GBM by suppressing angiogenesis by inducing mitochondrial depolarization and apoptosis [248]. Gambogic acid was reported to decrease Aβ accumulation by up-regulating the expression of HO-1 (Heme oxygenase-1) [249,250,251].

### 7.5. Flavonoid

Natural flavonoids, including kaemferol, myricetin, and quercetin, attach to the ATP-binding pocket of NTD and impede ATP binding, thereby degrading client protein and maintaining the proteostasis network [252,253,254] and by inhibiting P13K/Akt/mTOR signaling and enhancing LC3-II expression to induce autophagy [255,256]. Interestingly, quercetin (25 μM) inhibits the migration and growth of GBM cells by downregulating IL-6/STAT3 signaling [257]. Kaempferol inhibits the proliferation and migration of GBM cells in a ROS-dependent manner. In addition, Kaempferol (50 μmol/L) also enhances the chemosensitivity of doxorubicin against GBM by increasing ROS-mediated toxicity and reducing doxorubicin efflux [258]. Zhao et al. showed that myricetin (10–20 μM) could reduce GBM cell motility and invasion by inhibiting the formation of lamellipodia and focal adhesion [259]. Furthermore, by decreasing the short isoform of FLIP (FLICE-like inhibitory protein) and Bcl-2, myricetin (150 μM) makes malignant GBM cells more vulnerable to TRAIL-mediated apoptosis [260].

Chakrabarti et al. identified the anti-tumor activities of silibinin and luteolin in GMB cell lines, which showed inhibition by decreasing autophagy and inducing miR-7-1-3p expression and apoptosis [261]. Besides, it is also reported that the malignant phenotypes of GBM cells are disturbed by luteolin (30 µM) because it blocks Musashi1 from binding to RNA [262]. Furthermore, PI3K/AKT activation, Cdc42 (Cell Division Cycle 42) protein production, and proteasome-mediated degradation are all mechanisms by which luteolin inhibits GBM cell migration [263,264,265]. In GBM cells and GBM stem cells, luteolin and silibinin acted synergistically to inhibit cell migration and invasion and cause death at 20 mM and 50 mM, respectively [266]. Silibinin increased the chemosensitivity of trametinib and dabrafenib when combined as a personal treatment for the GBM patient with BRAF V600E mutation [267]. When treated with arsenic trioxide (ATO), silibinin synergically increased proliferation and decreased gelatinase A and B activities, metabolic activity, and cell proliferation [268,269]. Silibinin (50, 100, and 150 μM) promoted apoptosis and autophagy in GBM cells, which may have been caused by the simultaneous suppression of mTOR and YAP (yes-associated protein) [270]. Both silibinin and luteolin are reported to provide neuroprotection against various neuronal injuries [271]. Cortical neurons are particularly vulnerable to oxidative stress and cerebral ischemia-reperfusion injury, and silibinin (1 and 10 μM) was reported to protect them against mitochondrial damage and autophagy-induced cell death [271]. Furthermore, Liu et al. showed that silibinin (280 mg/kg) effectively protects dopaminergic neurons from neurodegeneration by reducing ROS, pro-inflammatory cytokines, NLRP3 (NLR family pyrin domain containing 3) activation, and α-Syn aggregation in the MPTP-treated mice model [272]. On the other hand, luteolin can inhibit oxidative stress, microglial and astrocyte reactivity, neuroinflammation, and disease severity in various neurodegenerative conditions, such as PD, AD, and MS [273,274,275,276,277,278]. Both silibinin [279] and luteolin [280] are identified as novel inhibitors of Hsp90.

Apigenin, a flavonoid found in plants, is reported to inhibit Hsp90 function by directly disrupting the Hsp90/Cdc37 complex and reducing the proliferation and migration of cancer cells at 60 μM [281]. Accumulating studies showed that apigenin (50 μM) could inhibit GBM cell proliferation and migration by inducing oxidative stress [282], cell cycle arrest, and regulating c-Met signaling at 25 μM [283], and apoptosis pathway [180,284]. Wang et al. reported the synergetic effect of apigenin in combination with TMZ against TMZ-resistant GBM cells by increasing the chemosensitivity of TMZ by decreasing MMP-2 and 9, BCL-2, AKT phosphorylation, and cyclin D1 [285]. Apigenin alleviates neuronal injuries by reducing oxidative stress, neuroinflammation, and NMDA (*N*-methyl-*D*-aspartate)-induced calcium excitotoxicity [286,287,288,289,290,291].

Chetomin is another natural anticancer compound, usually found in *Chaetomium globosum*, and is also known for its Hsp90 inhibition activity [292]. Chetomin can reduce HIF-1α in GBM cells and increase the radiation sensitivity in radiotherapy [293,294]. On the other hand, Chetomin (10 uM) reduces using mutated LRRK2 (Leucine-rich repeat kinase 2) mediated toxicity in the Drosophila model, which is a shared basis of autosomal dominant familial PD [295].

## 8. Conclusions and Future Prospective

Given disease complexity and heredity, many complex diseases share similar pathways and molecular targets which are common to each other, and consequently, targeting drugs on these pathways shares broad-spectrum activity [296]. As an illustration of the therapeutic influence of anticancer drugs on NDDs, consider that patients with breast cancer who undergo chemotherapy have a significantly reduced risk of developing AD in old age [297]. This finding suggests that anticancer drugs may help treat NDDs like AD due to the same signaling pathways between the two diseases [298]. Similarly, Hsp90 could be another potential example of a single target that can be targeted in multiple diseases, especially in both GBM and neurodegeneration.

Considering the aggressive nature of GBM and dismal prognosis, it is essential to investigate any or all factors that may be allowing these cells to survive despite therapy. As discussed above, Hsp90 and its client proteins have become validated as a novel drug target for GBM and other cancer treatments in both single and combination therapy since Hsp90 involves several molecular signalings of tumorigenesis, including apoptosis, proliferation, angiogenesis, migration, invasion, and metastasis. Similarly, many of the Hsp90 client proteins are involved in maintaining the degenerative condition in the neuron during any pathological insult. Indeed, targeting Hsp90 in neurodegeneration provides neuroprotection in dual ways, such as reducing the accumulation of misfolded protein aggregates by enhancing chaperone functions and inhibiting abnormal neuronal protein activity through alleviating protein hyperphosphorylation and consequent aggregation. These reports suggest that Hsp90 inhibition could improve the survivability of GBM patients while maintaining significant neuroprotection as a common target of cancer and neurodegeneration.

More than 50 clinical trial studies have been conducted to treat various cancers using Hsp90 inhibitors, but none of the studies has been focused on GBM or neurodegenerative diseases. However, combining Hsp90 inhibitors with standard GBM therapies like TMZ or radiation shows promise as a therapeutic strategy, with encouraging outcomes in in vitro/in vivo studies, suggesting further studies in various clinical settings.

Many clinical studies using Hsp90 inhibitors are also halted in the phase 1 trials due to toxicity, side effects, and reduced efficacy. As the therapeutic benefits of Hsp90 inhibitors depend on the non-toxic doses, an optimum dose should be determined with maximum effect, yet non-toxic, BBB penetrating and can use in long-term administration schedules. This requires many preclinical studies and more drug development efforts utilizing existing drugs and potential natural products.

From identifying the first Hsp90 inhibitors, natural products have been a promising source for developing potential inhibitors for Hsp90, from where several novels fully and semi-synthetic Hsp90 inhibitors are developed and advanced in clinical trials. As discussed above, many natural compounds targeting Hsp90 are promising in GBM and various neuronal injuries. Thus, research should focus more on the clinical trial of potential natural compounds, such as Hsp90 inhibitors, for treating GBM and neurodegeneration. Moreover, scaffolds of potential natural products could be subjected to design new promising derivatives further with the help of computational analysis. Since pharmacokinetics is an essential factor for natural products, especially for the compound targeting CNS, these findings emphasize the necessity of further studies on designing and improving the bioavailability of natural product-based Hsp90 inhibitors with optimal efficacy in both GBM and neurodegeneration.

It should also be acknowledged that not all of the Hsp90 client kinases are tumor-promoting factors, and some have rather growth-suppressive functions, like DAPK (death-associated protein kinase) and LKB1 (liver kinase B1), which inhibit cell proliferation [299]. Considering this, it is essential to target the client proteins specifically related to tumor promotion, or maintaining a careful balance between them is essential to focus on future studies. Although much preclinical evidence, as highlighted in this review, showed definitive evidence of Hsp90 regulation and its inhibition by the individual model of GBM and neurodegeneration, studies on the combined model of GBM and neurodegeneration or GBM-induced neurodegeneration remain undetermined and critical for the concurrent treatment of Hsp90 inhibitors in both conditions. More studies are also needed to elucidate the pathology mechanism of GBM following neurodegeneration, and the role of Hsp90 in this dynamics scenario should be investigated briefly. These insights provide a foundation for further research into ideas that might inform the development of new drugs for the concurrent treatment of GBM and neurodegeneration.
